# Impaired Barrier Function and Immunity in the Colon of Aldo-Keto Reductase 1B8 Deficient Mice

**DOI:** 10.3389/fcell.2021.632805

**Published:** 2021-02-12

**Authors:** Xin Wang, Ramina Khoshaba, Yi Shen, Yu Cao, Minglin Lin, Yun Zhu, Zhe Cao, Duan-Fang Liao, Deliang Cao

**Affiliations:** ^1^Department of Medical Microbiology, Immunology & Cell Biology, Simmons Cancer Institute, Southern Illinois University School of Medicine, Springfield, IL, United States; ^2^Department of Biotechnology, College of Science, University of Baghdad, Baghdad, Iraq; ^3^State Key Laboratory of Chinese Medicine Powder and Medicine Innovation in Hunan (incubation), Division of Stem Cell Regulation and Application, Hunan University of Chinese Medicine, Changsha, China

**Keywords:** aldo-keto reductase 1B8, intestinal epithelial cells, intestinal immunity, cytokines, AKT and ERK signaling pathways

## Abstract

Aldo-keto reductase 1B10 (AKR1B10) is downregulated in human ulcerative colitis (UC) and colorectal cancer, being a potential pathogenic factor of these diseases. Aldo-keto reductase 1B8 (AKR1B8) is the ortholog in mice of human AKR1B10. Targeted AKR1B8 deficiency disrupts homeostasis of epithelial self-renewal and leads to susceptibility to colitis and carcinogenesis. In this study, we found that in AKR1B8 deficient mice, Muc2 expression in colon was diminished, and permeability of colonic epithelium increased. Within 24 h, orally administered FITC-dextran penetrated into mesenteric lymph nodes (MLN) and liver in AKR1B8 deficient mice, but not in wild type controls. In the colon of AKR1B8 deficient mice, neutrophils and mast cells were markedly infiltrated, γδT cells were numerically and functionally impaired, and dendritic cell development was altered. Furthermore, Th1, Th2, and Th17 cells decreased, but Treg and CD8T cells increased in the colon and MLN of AKR1B8 deficient mice. In colonic epithelial cells of AKR1B8 deficient mice, p-AKT (T308 and S473), p-ERK1/2, p-IKBα, p-p65 (S536), and IKKα expression decreased, accompanied with downregulation of IL18 and CCL20 and upregulation of IL1β and CCL8. These data suggest AKR1B8 deficiency leads to abnormalities of intestinal epithelial barrier and immunity in colon.

## Introduction

Colorectal cancer (CRC) ranks at the third in newly diagnosed cancer cases and is the second leading cause of cancer death in U.S (Siegel et al., 2017). Ulcerative colitis (UC) that affects inner layer of large intestine and rectum is prevalent at about 286 per 100,000 in the U.S. (Ng et al., 2017). A variety of risk factors may contribute to UC and CRC development and progression, including genetics, intestinal immunity, and gut microbiota (Tariq and Ghias, 2016). Intestinal epithelium is the key node in the regulatory network of intestinal function and gut health. Intestinal epithelial cells (IECs) mediates gut immunity (Maloy and Powrie, 2011). In addition to being a physical barrier that separates lamina propria from luminal pathogens, IECs can sense and respond to environmental factors by producing cytokines, mucus, and antimicrobial peptides (AMPs), thus controlling intestinal homeostasis (Peterson and Artis, 2014). IEC defects may leads to intestinal permeability and translocation of luminal pathogens, stimulating aberrant immune response (Salim and Söderholm, 2011). In Ames dwarf mice with a missense mutation in Prop1 gene, retarded development of intestine deters intestinal innate and adaptive immunity (Wang et al., 2018), and in C57BL/6 mice, targeted disruption of aldo-keto reductase 1B8 (*AKR1B8*) gene disturbs self-renewal of colonic cryptic cells and leads to high susceptibility to dextran sulfate sodium (DSS)-induced colitis and associated carcinogenesis (Shen et al., 2015). Therefore, any genetic or hormonal factors that affect epithelial development and function may lead to a series of sequential abnormalities in epithelial cells and intestinal immunity, eventually causing UC and/or colorectal carcinoma (Hanahan and Weinberg, 2011).

Mouse *AKR1B8* is the ortholog of human aldo-keto reductase 1B10 (*AKR1B10*) (Joshi et al., 2010). AKR1B10 protein is primarily expressed in colon and small intestine (Cao et al., 1998; Joshi et al., 2010) and functions as a monomeric cytosolic enzyme with strong activity to α, β-unsaturated carbonyl compounds, protecting the host cells from carbonyl lesions (Yan et al., 2007; Wang et al., 2009; Zhong et al., 2009; Shen et al., 2011). AKR1B10 also mediates *de novo* synthesis of long chain fatty acids and membrane lipids, such as phosphatidylinositol 4,5-bisphosphate (PIP_2_) through regulating acetyl-CoA carboxylase-α (ACCA) stability (Ma et al., 2008). PIP_2_ is a critical signal molecule that mediates membrane-based signaling transduction, such as, PI3K/AKT and PKC/ERK pathways (Huang et al., 2018). Interestingly, AKR1B10 is lost and may pathogenically contribute to carcinogenesis in CRC (Zu et al., 2017). Data in microarray datasets (GSE39582) showed that AKR1B10 expression decreased in colon adenocarcinomas at all stages (Supplementary Figure 1A), and low expression of AKR1B10 was associated with reduced survival rate, being a potential prognostic marker in colorectal cancer (Taskoparan et al., 2017).

AKR1B10 is also downregulated in UC and colitis-associated colorectal cancer (CAC). Data from microarray datasets GSE38713 in GEO exhibited similar results (Supplementary Figure 1B). In UC, AKR1B10 expression decreased in both remitted and active UC. However, little is known of the mechanistic role of AKR1B10 deficiency in the development and progression of these human intestinal diseases. In mice, AKR1B8 deficiency leads to susceptibility to colitis and associated carcinogenesis. This is similar to the phenomenon in human cases, where AKR1B10 expression is diminished. In this study, therefore, *AKR1B8* knockout (–/–) mice were used as a model to investigate its role in intestinal epithelial barrier and immunity and the data indicated the importance of AKR1B8 in the intestinal epithelial integrity and innate and adaptive intestinal immunity, suggesting its potential pathogenic contributions in the intestinal diseases, such as UC and CRC.

## Materials and Methods

### Ethics Statement

Animal protocols were approved by Southern Illinois University School of Medicine Laboratory Animal Care and Use Committee (LACUC; Springfield, IL).

### Animals

Mice were housed in the animal facility at Southern Illinois University School of Medicine at 24°C ± 0.5°C, 50% ± 10% humidity with 12 h of light from 8:00 am to 8:00 pm and free access to regular diet and tap water. Heterozygous AKR1B8 knockout (+/–) C57BL/6 mice (Shen et al., 2015) were used to produce homozygous *AKR1B8* knockout *(–/–*) (KO) and littermate wild-type (WT) mice for experimental studies.

### *In vivo* Intestinal Permeability Assay

Intestinal permeability was measured by oral administration of FITC-dextran (40,00 MW; TdB Consultancy) (0.5 g/kg body weight) to mice for 24 h. At indicated time points, mice were euthanized; mesenteric lymph nodes (MLN) and livers were excised and embedded with OTC for cryostat section using a standard procedure (Hanahan and Weinberg, 2011).

### Epithelial Crypt, Single Epithelial Cell, and Lamina Propria Leucocyte Isolation

Epithelial crypts (ECs) and lamina propria cells were isolated from colon as previously reported (Wang et al., 2018). Briefly, ECs were collected using HBSS buffer supplemented with 2% FBS, 5 mM EDTA and 1 mM DTT (American Bioanalytical). Single epithelial cell suspensions were made by digestion of crypts in HBSS containing 0.5 mg/ml of dispase II (Roche) at 37°C for 10 min with intermittent shaking. Lamina propria leukocytes (LPLs) were isolated by digestion of lamina propria tissues in Dulbecco's PBS with 10% FBS, 0.5 mg/ml dispase II, 0.5 mg/ml collagenase D (Roche), and 100 U DNase I (Sigma) at 37°C for two consecutive 20 min. LPLs were then recovered by Percoll gradient centrifugation at 1,000 g for 20 min.

### Mesenteric Lymph Node and Spleen Cell Isolation

Mesenteric lymph nodes (MLN) and spleens were cut into small pieces and then squeezed with syringe tips. Single cell suspensions were collected from flow-through of the nylon cell strainer. Red blood cells were removed using lysis buffer (Biolegend).

### Cell Staining and Flow Cytometry Analysis

Cells were blocked with anti-mouse CD16/CD32 antibody (Clone 93, BioLegend) and then stained with appropriate cell surface marker antibodies, followed by flow cytometry analysis (Harrington et al., 2005). To assess intracellular IFNγ, IL17, IL4, IL10, and IL22, cells were stimulated with 50 ng/ml Phorbol 12-Myristate 13-Acetate (PMA), followed by fixation, permeabilization and staining with appropriate antibodies (Harrington et al., 2005). A True-Nuclear Transcription Factor Buffer Set (BioLegend) was used for intracellular Foxp3, and 7-amino-actinomycin D (7AAD) was applied for dead cell exclusion. Flow cytometry analysis was performed using an Accuri C6 or BD FACS Aria II flow cytometer, and data were analyzed using a FlowJo software (Tree Star). Supplementary Figures 2, 3 show the gating strategies of tested cells. All antibodies were purchased from Biolegend. CD3 (100235), CD4 (100413), CD8 (100705), CD11B (101229), CD11C (117305), Ly6G (127613), CD45(103107), IFNγ (505809), IL17 (506907), IL4 (144807), IL10 (505013), IL22 (516404).

### Immunofluorescent and Mast Cell Staining

Immunofluorescent assays with 4', 6-diamidino-2-phenylindole (DAPI) for nuclear staining were conducted as previously described (Hall et al., 2013). Mast cells were assessed using paraffin-embedded sections stained with 0.5% toluidine blue (pH 0.3) and eosin (Sigma). After mounting, slides were reviewed and photographed under a microscopy (OLYMPUS DP73).

### Real-Time RT-PCR

Total RNA extraction and quantitative real-time RT-PCR with SYBR green qPCR mixture were conducted as previously described (Shen et al., 2015). See Supplementary Table 1 for gene-specific primer sequences.

### Western Blot

Soluble protein preparation and Western blotting were conducted as previously described (Shen et al., 2015). Antibodies are purchased from Cell Signaling Biotechnology. AKT (9272S), p-AKT (4060S/13038S), ERK (4695T), p-ERK (9101S), p-p90RSK (11989S), p-MSK1 (9595T), β-actin (4970S), IKKα (61294S), IKKβ (8943S), p-IKKα/β (2697S), p-IKBα (9246S), t-IKBα (9242S), p-P65 (3033S/ 3037), and P65 (8242T).

### Statistical Analysis

Unpaired two-tailed Student *t*-test were applied for statistically significant analysis with *p* < 0.05 as statistical significance. All statistical tests were done using GraphPad software (San Diego, CA).

## Results

### Increased Colon Mucosal Permeability in *AKR1B8 –/–* Mice

**Figure 1:**
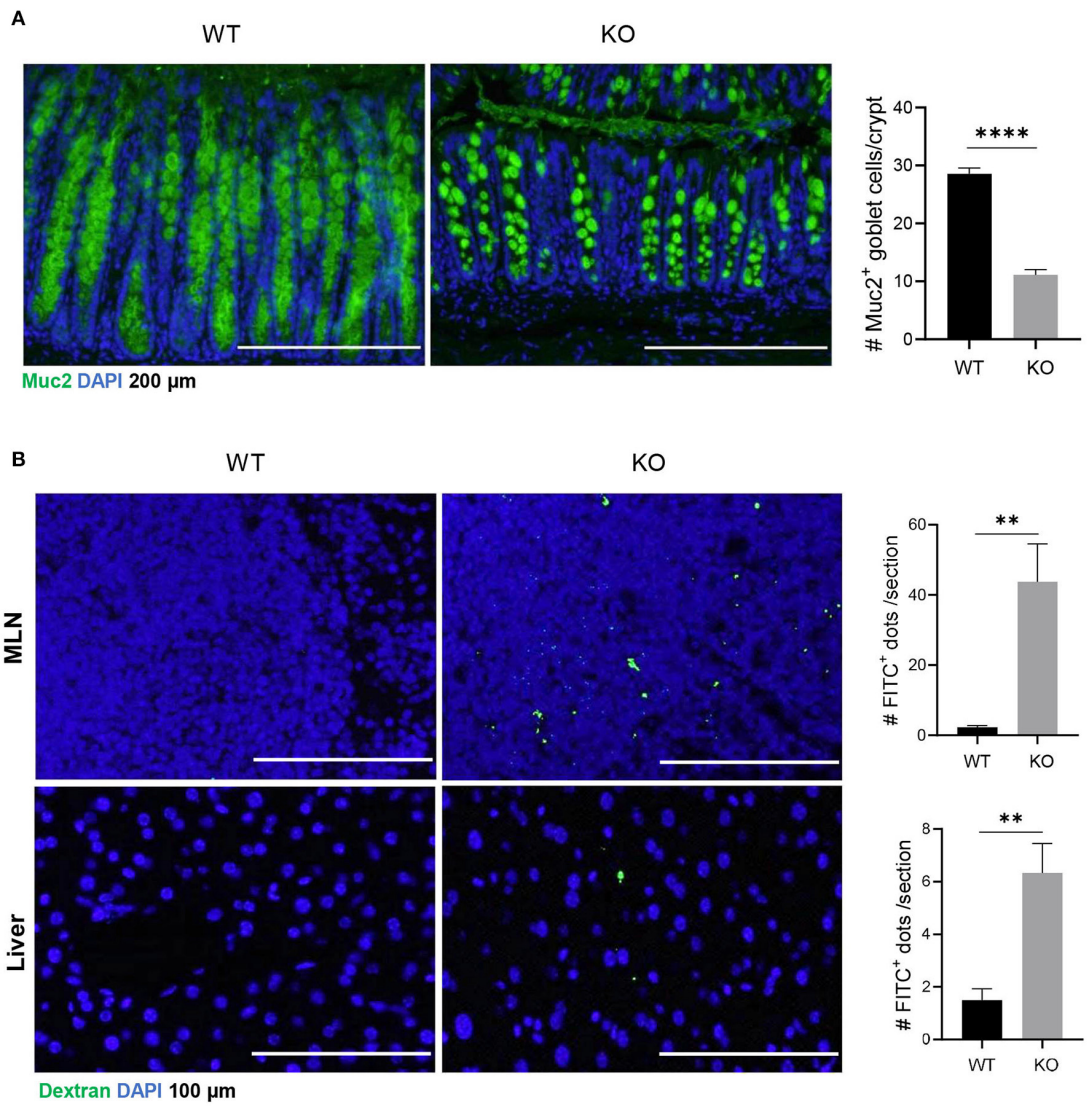
Decreased muc2 expression and increased permeability of colonic epithelium in *AKR1B8 –/–* mice. **(A)** Muc2 expression (green) in colon, detected by immunofluorescent staining. DAPI (blue) was used for nuclear staining. Scale bars, 200 μm. Muc2-producing goblet cells per crypt were counted (*right*). Data indicate mean ± SEM. **(B)** FITC-Dextran in mesenteric lymph nodes (MLN, *upper panel*) and liver (*lower panel*). FITC signals per section were quantitated with an ImageJ software (*right*). Nuclei were stained with DAPI (Blue). Scale bars, 100 μm. WT, wild-type; KO, *AKR1B8 –/–*. *P < 0.05; **P < 0.01; ***P < 0.001; ****P < 0.0001.

Muc2 is a major component of luminal mucus that functions as the first barrier to physically separate luminal pathogens from colonic epithelium (Birchenough et al., 2015). Our results showed that in AKR1B8 deficient epithelium, Muc2 expression was diminished and Muc2 expression goblet cells decreased (Figure 1A). Oral administration of mice with FITC-labeled dextran showed that the FITC-dextran appeared in MLN and livers within 24 h in *AKR1B8 –/–* mice, but not in littermate wild type (WT) controls (Figure 1B). These data indicate the impaired barrier function and increased mucosal permeability in the colon of *AKR1B8 –/–* mice.

### Enhanced Infiltrations of Neutrophils and Mast Cells in the Colon of *AKR1B8 –/–* Mice

**Figure 2:**
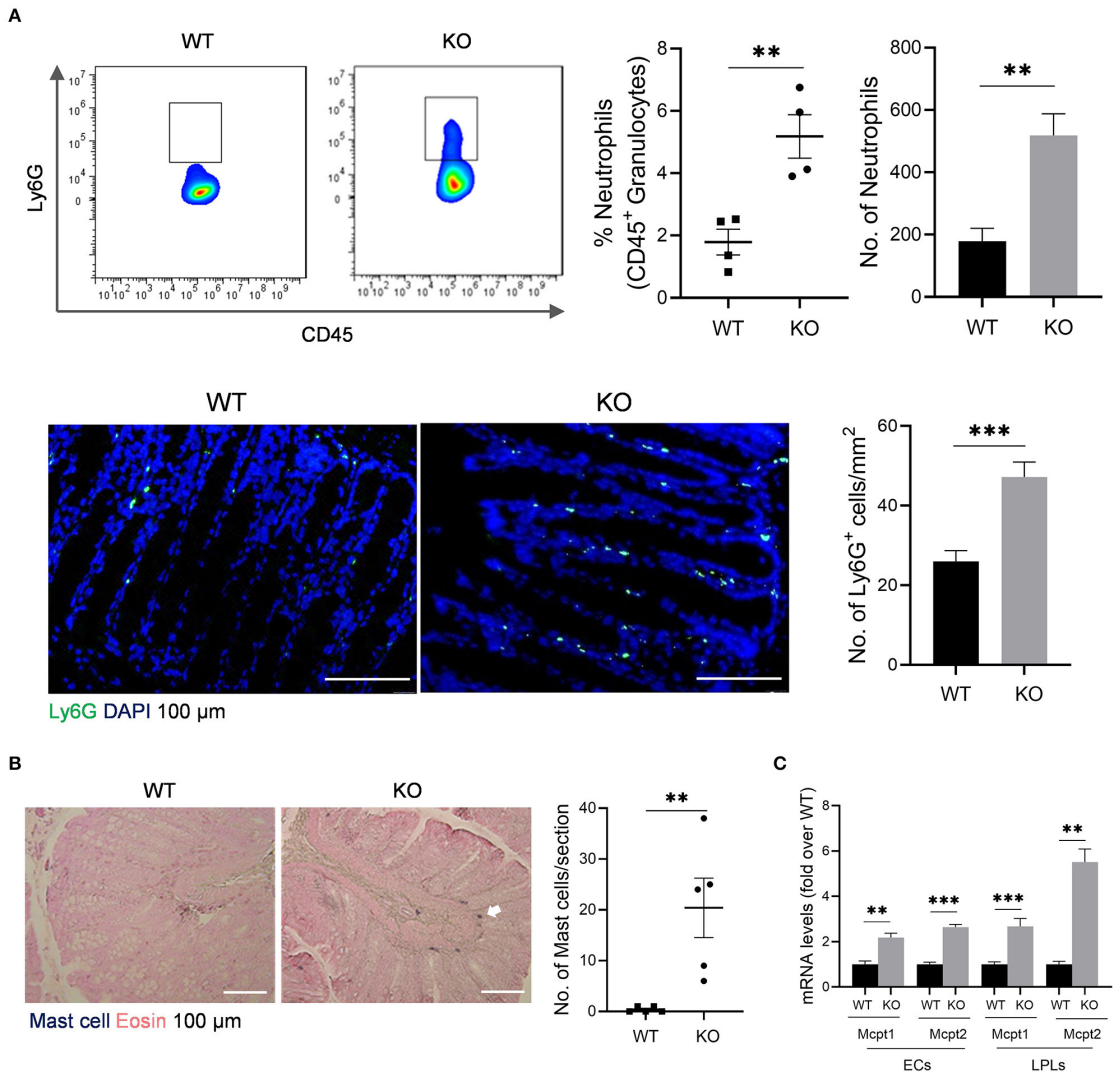
Granulocyte infiltrates in the colon of *AKR1B8 –/–* mice. **(A)** Neutrophils. *Upper panel*: flow cytometry. Lamina propria leukocytes (LPLs) were isolated from colons and subjected to flow cytometry analysis as described in Materials and Methods. *Left*, flow cytometry images; *middle*, percentage of neutrophils in CD45^+^ granulocytes; *right*, number of neutrophils in 10^4^ CD45^+^ granulocytes. *Lower panel*: immunofluorescent staining of neutrophils. *Left*, FITC-labeled Ly6G and DAPI staining images, scale bar: 20 μm; *right*, Ly6G positive cell number per mm^2^. **(B)**. Mast cells. *Left*, toluidine blue and eosin staining images, scale bar: 100 μm; white arrow: mast cells. *Right*, mast cell number per section. **(C)**. Expression levels of mast cell protease 1 and 2 in colonic epithelial crypts (cECs) and isolated LPLs. Data shown are quantitative RT-PCR results. WT, wild-type; KO, *AKR1B8 –/–*. Data denote mean ± SEM of four mice per group. *P < 0.05; **P < 0.01; ***P < 0.001, independent samples t-test.

The impaired barrier function of colon mucosa may allow luminal pathogen invasion and thus activates inflammatory response. Neutrophils are the first responders in host defense. Our data showed that neutrophils increased in colonic lamina propria (cLP) of *AKR1B8 –/–* mice compared to wild type littermates (Figure 2A, *upper panel*). Immunofluorescent assays confirmed this finding (Figure 2A, *lower panel*). Mast cells also increased in the colon of *AKR1B8 –/–* mice (Figure 2B), and mast cell-specific protease-1 (mMCP-1) and protease-2 (mMCP-2) expression increased by over 2 times in isolated crypts and LPLs (Figure 2C). These data suggest that *AKR1B8* deficiency induces infiltration of neutrophils and mast cells in the colon mucosa.

### Numerical and Functional Defects of γδT Cells in the Colon of *AKR1B8 –/–* Mice

**Figure 3:**
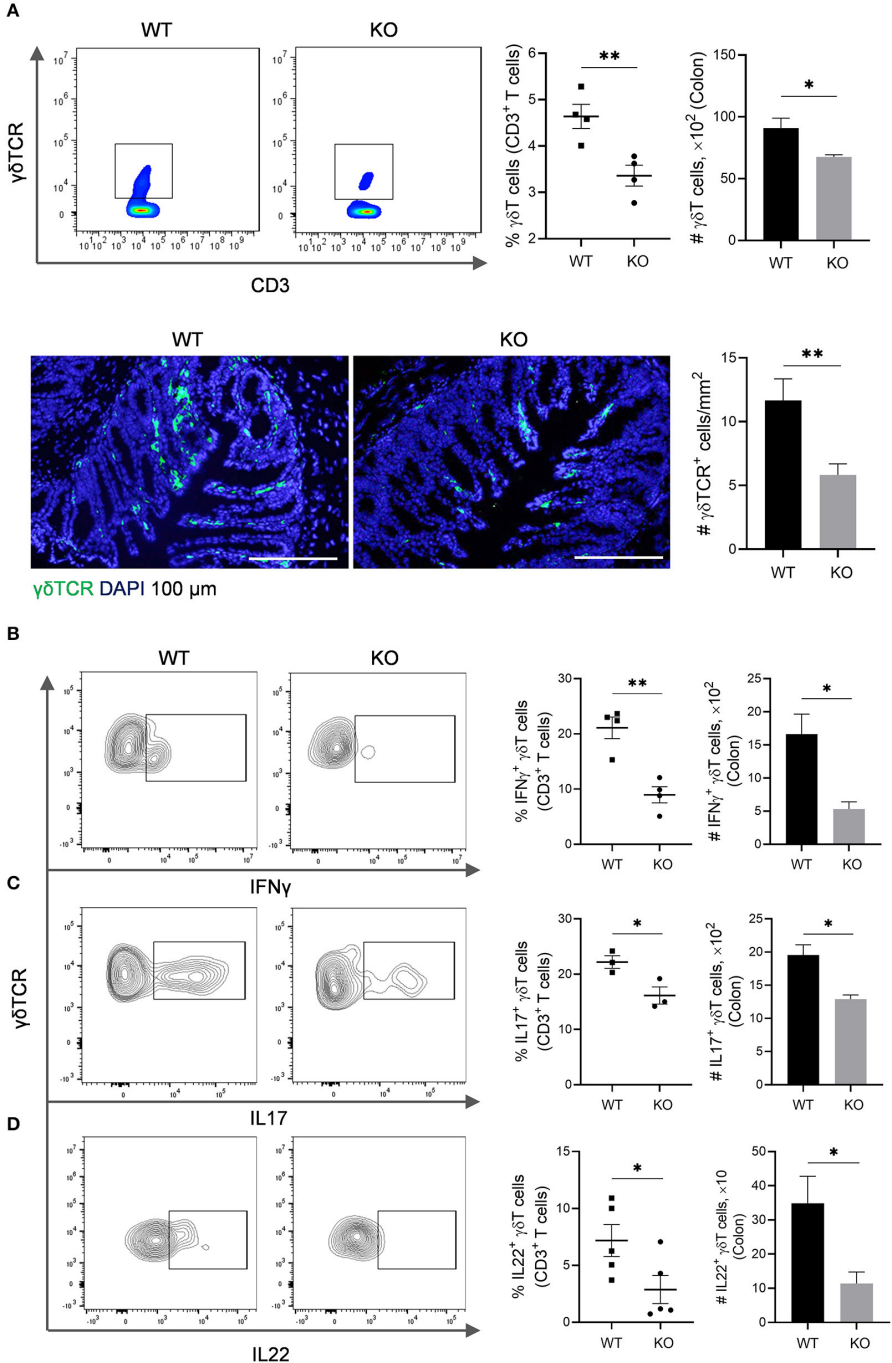
γδT cells in the colon of *AKR1B8 –/–* mice. **(A)** γδT cell number. *Upper panel*: Flow cytometry. Lamina propria leukocytes (LPLs) were isolated from colons and subjected to flow cytometry analysis as described in Materials and Methods. *Left*, flow cytometry images; *middle*, percentage of γδT cells in CD3^+^ T cells; *right*, number of γδT cells in 10^4^ CD3^+^ T cells. *Lower panel*: Immunofluorescent staining of γδT cells. *Left*, FITC-labeled γδTCR and DAPI staining images, scale bar: 20 μm; *right*, γδTCR positive cell number per mm^2^. **(B**–**D)** IFNγ, IL17, and IL22-producing γδT cells. LPLs were isolated from colon and stimulated with PMA and ionomycin in presence of protein transport inhibitor for 5 h, followed by staining for CD3, γδTCR, and then IFN-γ, IL17, or IL22. *Left*, flow cytometry images; *middle*, percentage of IFNγ, IL17, and IL22-producing γδT cells in total γδT cells*; right*, number of IFNγ, IL17, and IL22-producing γδT in 10^4^ γδT cells. WT, wild-type; KO, *AKR1B8 –/–*. Data indicate mean ± of SEM of four mice per group. *P < 0.05; **P < 0.01.

The γδT cells link innate and adaptive immunity, functioning in antigen presentation, cytokine production and bacterial clearance (Bonneville et al., 2010). We further evaluated γδT cells in the colon of *AKR1B8 –/–* mice. Results showed that γδT cells decreased in cLP of *AKR1B8 –/–* mice (Figure 3A, *upper panel*). This was confirmed by immunofluorescent staining assays (Figure 3A, *lower panel*). γδT cells function by secretion of IFNγ, IL17, and IL22 (Bonneville et al., 2010). Our results showed the γδT cells that produce IFNγ (Figure 3B), IL17 (Figure 3C), and IL22 (Figure 3D) all decreased. These data indicate the impaired development and function of γδT cells in the colon of *AKR1B8 –/–* mice.

### Impaired Antigen Presentation in the Colon and MLN of *AKR1B8 –/–* Mice

**Figure 4:**
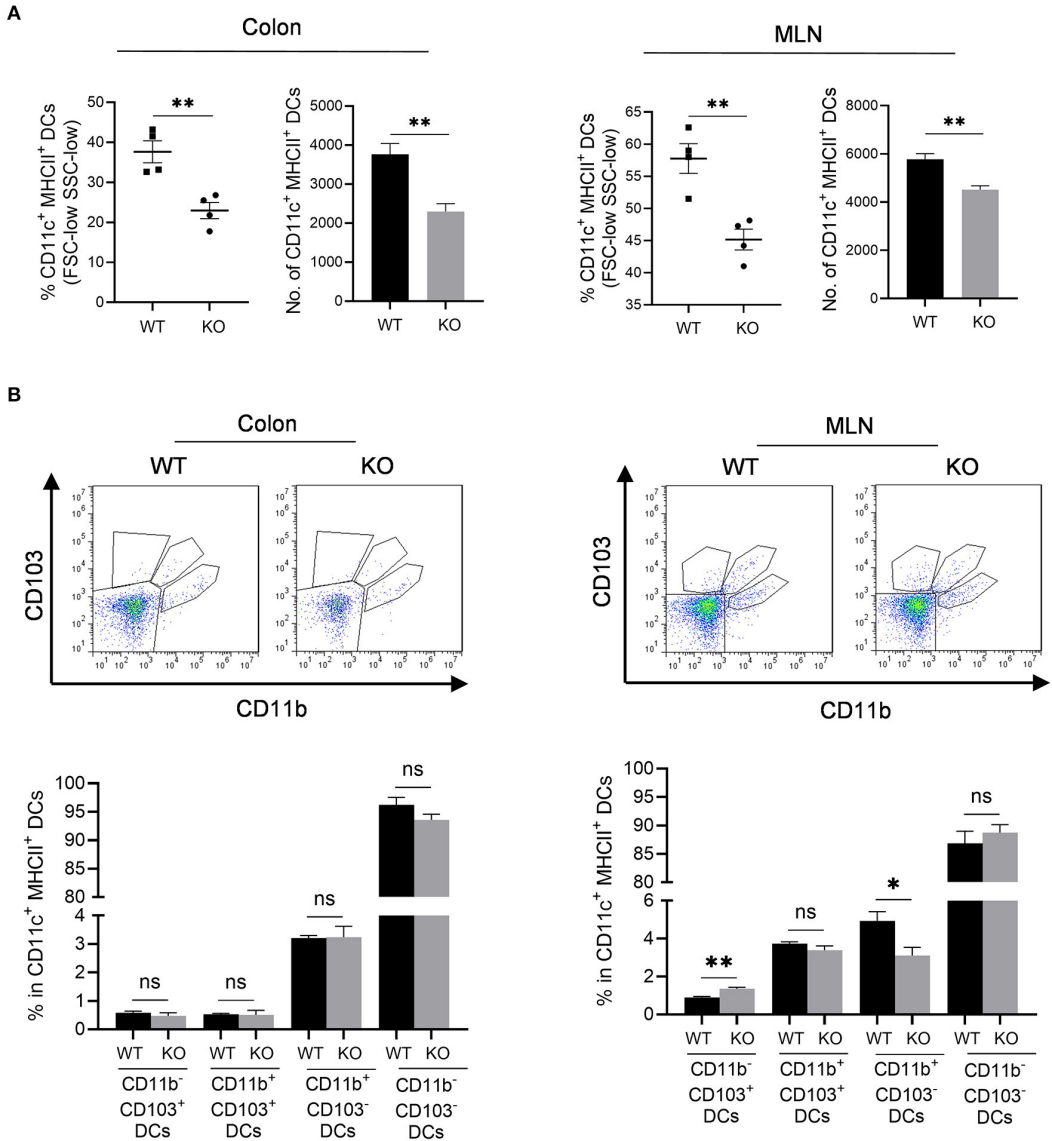
Antigen presentation cells in colon and MLN of *AKR1B8 –/–* mice. Lamina propria leukocytes (LPLs) and mesenteric lymph node (MLN) cells were isolated and analyzed by flow cytometry as described in Materials and Methods. **(A)** MHCII^+^ dendritic cells in colonic lamina propria and MLN. *Left*, percentage of CD11c^+^ MHCII^+^ DCs in FSC-low SSC-low mononuclear cells; *right*, number of CD11c^+^ MHCII^+^ DCs in 10^4^ FSC-low SSC-low mononuclear cells. **(B)** DC subtypes in colon and MLN. *Upper*, flow cytometry images; *lower*, percentage of CD103^+^ DC subsets in CD11c^+^ MHCII^+^ DCs. WT, wild-type; KO, *AKR1B8 –/–*. Data indicate mean ± SEM of four mice per group. *P < 0.05; **P < 0.01.

We then assessed professional antigen presentation cells, dendritic cells (DCs) in the colon and mesenteric lymph nodes (MLN) of *AKR1B8 –/–* mice (Supplementary Figure 3). Results showed that CD11c^+^ MHCII^+^ DCs significantly decreased in colon and MLN (Figure 4A). MHCII^+^ DCs are migratory, which develop in colon and migrate to MLN to present antigens to naïve T cells (Guermonprez et al., 2002). We thus investigated subtypes of MHCII^+^ DCs using CD11b and CD103 markers. Results showed that CD11b and CD103 subtypes of DCs were not notably changed in colon (Figure 4B, *left panel*), but CD11b^−^ CD103^+^ DCs increased while CD11b^+^ CD103^−^ DCs decreased in the MLN of *AKR1B8 –/–* mice (Figure 4B, *right panel*). These data suggest that AKR1B8 deficiency disturbs the development of MHCII^+^ DCs in the colon and MLN.

### Decreased T Helper Cells but Increased Cytotoxic T Cells in Colon and MLN of *AKR1B8 –/–* Mice

**Figure 5:**
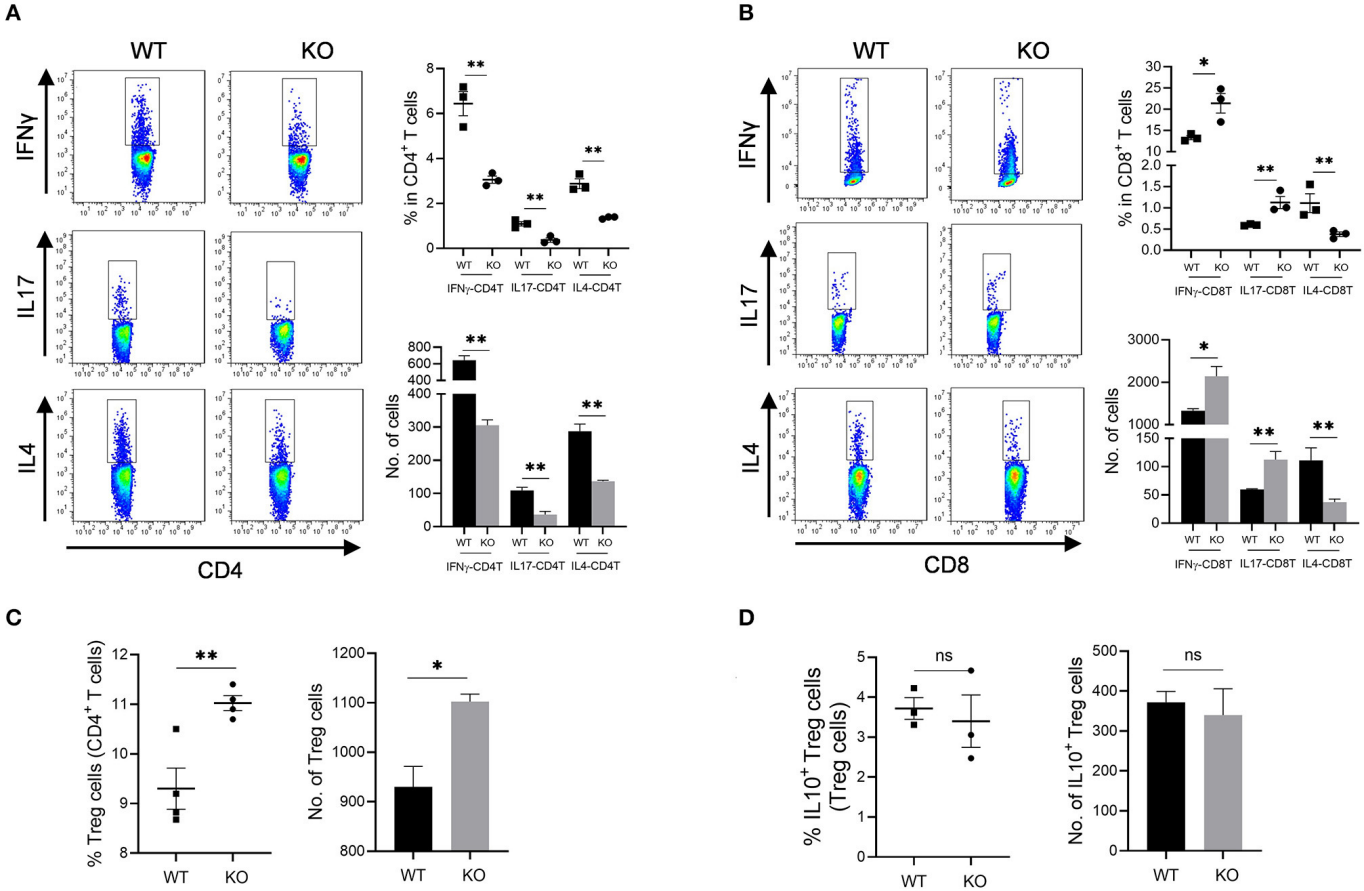
Adaptive CD4T and CD8T cells in MLN of *AKR1b8 –/–* mice. Mesenteric lymph node (MLN) cells were isolated and analyzed by flow cytometry as described in Materials and Methods. **(A)** IFNγ, IL17, and IL4-producing CD4T helper cells. *Left panel*, flow cytometry images of IFNγ-CD4T (Th1), IL17-CD4T (Th17), and IL4-CD4T (Th2) cells; *right upper*, percentage of IFNγ, IL17, and IL4-CD4T in total CD4T cells, *right lower*, number of IFNγ, IL17, and IL4-CD4T cells in 10^4^ CD4T cells. **(B)**. IFNγ, IL17 and IL4-producing cytotoxic CD8T cells. *Left panel*, flow cytometry images of IFNγ-CD8T (Tc1), IL17-CD8T (Tc17), and IL4-CD8T (Tc2) cells; *right upper*, percentage of IFNγ, IL17 and IL4-CD8T in CD8T cells, *right lower*, number of IFNγ, IL17, and IL4-CD8T cells in 10^4^ CD8T cells. **(C)** CD4^+^ Treg cells. *Left*, percentage of CD4^+^ Treg cells in CD4T cells; *right*, number of Treg cells in 10^4^ CD4T cells. **(D)** IL10-producing Treg cells. *Left*, percentage of IL10-producing Treg cells in total Treg cells; *right*, number of IL10-producing Treg cells in 10^4^ total Treg cells. WT, wild-type; KO, *AKR1B8 –/–*. Data represent mean ± SEM form four mice per group. *P < 0.05; **P < 0.01.

Increased mucosal granulocyte infiltrates and abnormal γδT and DC cells in the colon and MLN of *AKR1B8 –/–* mice further impaired adaptive immunity. Our data showed that PMA-stimulated cytokine producing T cells altered remarkably in *AKR1B8 –/–* mice (Figure 5) although the number of total T cells (CD3T) and subtypes (CD4T and CD8T) were not notably changed (Supplementary Figure 4A). In the MLN, IFNγ, IL4 and IL17-producing CD4T (i.e., Th1, Th2, and Th17) cells all decreased (Figure 5A), but IFNγ and IL17-producing CD8T (Tc1 and Tc17) cells increased while IL4-producing CD8T (Tc2) cells decreased (Figure 5B). In the colon of *AKR1B8 –/–* mice, IFNγ and IL17-producing CD4T cells also decreased, but IL4-producing CD4T cells (Supplementary Figure 4B) and IFNγ, IL17 and IL4-producing CD8T cells (Supplementary Figure 4C) were not altered. CD4T and CD8T cells are developed and matured in MLN and then disseminated into colon mucosa. This may explain the differential displays of T cells in MLN and colon.

We further investigated Treg cells in *AKR1B8 –/–* mouse colon. Results showed Foxp3^+^ Treg cells for immune tolerance and suppression increased in MLN of *AKR1B8 –/–* mice (Figure 5C) and tended to increase in colon (*p* = 0.065) (Supplementary Figure 4D), but IL10-producing Treg cells were not notably changed in MLN and colon (Figure 5D and Supplementary Figure 4E), indicating a balanced steady status. Together these data suggest that AKR1B8 deficiency alters T cell immunity with decreased CD4T but increased CD8T activity.

### Inhibition of AKT, ERK, and NF-κB Signaling Cascades in cECs of *AKR1B8 –/–* Mice

**Figure 6:**
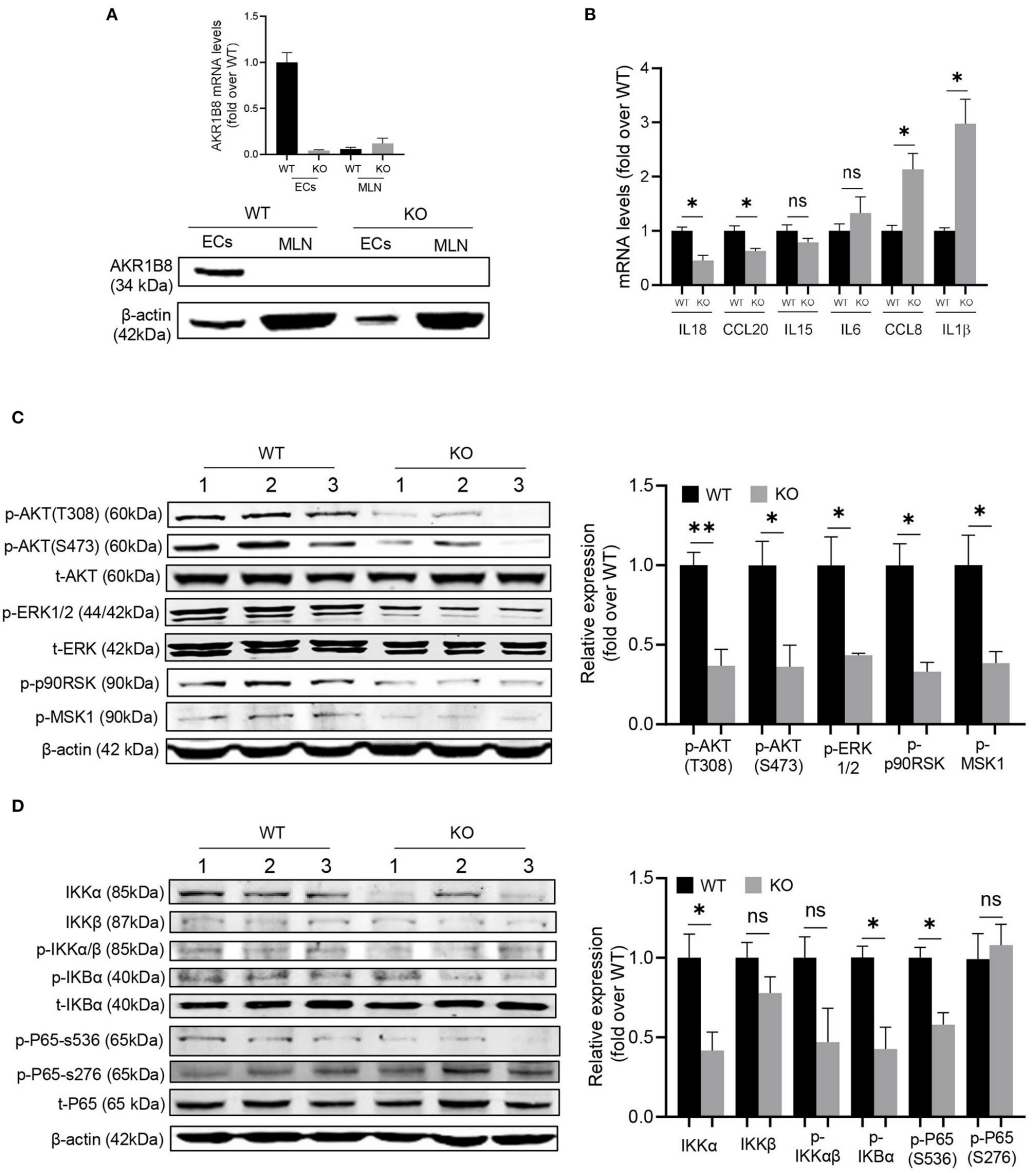
Molecular mechanisms of intestinal immune defects in *AKR1B8 –/–* mice. **(A)** AKR1B8 expression in colonic epithelial cells (ECs) and immune cells from mesenteric lymph nodes (MLN). RNA and proteins were extracted for qRT-PCR (*upper panel*) and Western blot (*lower panel*) analyses as described in Materials and Methods. **(B)** Expression of IL18, CCL20, IL15, IL6, CCL8, and IL1β cytokines in ECs. Data indicate qRT-PCR results as described in Materials and Methods. **(C)** AKT and ERK signaling activity. The p-AKT (Ser473), p-AKT (Thr308), t-AKT, p-ERK (1/2), t-ERK, p-p90RSK and p-MSK1 proteins were analyzed by Western blot as described in Materials and Methods. β-actin was used as an internal control. *Left panel*, representative images; *right panel*, quantitative data of band intensity by Image J software. Data were normalized by respective total proteins. **(D)** NF-κB signal activity. IKKα, IKKβ, p-IKKα/β, p-IKBα, t-IKBα, p-P65 (S536), p-P65 (S276), and t-P65 protein levels were analyzed by Western blot. *Left panel*, representative images; *right panel*, quantitative data of band intensity by Image J software. Data of phosphorylated proteins were normalized by respective total proteins. Data of IKKα and IKKβ were normalized by β-actin. WT, wild-type; KO, *AKR1B8 –/–*. Data represent mean ± SEM of three to four mice per group. *P < 0.05; **P < 0.01.

This *AKR1B8 –/–* mouse strain is conventional, and thus we first examined AKR1B8 expression in colon epithelial cells (cECs) and immune cells from MLN. Results showed that AKR1B8 was highly expressed in cECs of wild type mice, but nullified in *AKR1B8 –/–* mice (Figure 6A); AKR1B8 was not expressed in immune cells (Figure 6A). These data indicate that *AKR1B8* knockout would not have direct effects on immune cells as AKR1B8 is not expressed; and the intestinal immune defects in *AKR1B8 –/–* mice may be derived from cEC deficiency. We further examined the expression of immunoregulatory cytokines in cECs and data showed that immunoregulatory IL18 and CCL20 were downregulated while IL1β and CCL18 were upregulated in cECs of *AKR1B8 –/–* mice (Figure 6B).

We then explored the signaling transduction that results in deficiency and abnormal expression of cytokines in cECs. AKR1B10 (AKR1B8 in mice) promotes long chain fatty acid and membrane lipid synthesis and activates PIP_2_ lipid messenger system (Ma et al., 2008; Huang et al., 2018). PIP_2_ mediates PI3K/AKT and PKC/ERK signaling transduction (Luo et al., 2003). We thus evaluated AKT and ERK activity in cECs. Data showed that p-AKT (T308 & S473) and p-ERK decreased in *AKR1B8 –/–* cECs (Figure 6C); ERK downstream effectors p-RSK90 and p-MSK1 also decreased (Figure 6C).

NF-κB is a master signaling cascade that regulates cytokine expression and immune response; IκB is a main negative regulator that holds NF-κB homo- or heterodimers in cytosol. AKT phosphorylates Thr23 of IKKα and activates IKKα, which in turn phosphorylates IκBα and triggers NF-κB nuclear translocation (Romashkova and Makarov, 1999). ERK also activates NF-κB signaling through phosphorylation and activation of RSK90 and MSK1 that in turn phosphorylate p65/RelA at Ser536 (Wang et al., 2010). Our data showed that in cECs of *AKR1B8 –/–* mice, p-IKKα/β, p-IKBα and p-p65 (Ser536) all decreased (Figure 6D). Together our data suggest that AKR1B8 deficiency inhibits AKT and ERK signaling activity in cECs that in turn impedes the NF-κB signaling cascade, deregulating immunoregulatory cytokine expression and intestinal immunity.

## Discussion

Muc2 is produced by goblet cells and secreted to line on the epithelial layer of intestine, where it functions as a physical barrier to block luminal pathogen invasion. Decreased Muc2 production associates with UC in humans (Van der Sluis et al., 2006), and *Muc2 –/–* causes spontaneous colitis in mice (Van Klinken et al., 1999), suggesting its critical role in intestinal health. AKR1B8 deficiency disrupts self-renewal and injury repair of colon epithelial cells and thus the mice were susceptibility to DSS-induced colitis and associated carcinogenesis (Shen et al., 2015). This study investigated the function of AKR1B8 in intestinal epithelial barrier and immunity under naïve status. We found that Muc2 expression in goblet cells decreased and mucosal permeability increased; the intestinal immunity was re-shaped.

Innate immune cells respond as the first line and function for extracellular clearance of pathogens. Results showed that neutrophils and mast cells increased in the colon of *AKR1B8 –/–* mice. Mast cell proteases, mCPT1 and mCPT2, are predominant products expressed by mast cells and function for extracellular microbial or parasite clearance (Urb and Sheppard, 2012). The increased expression of mCPT1 and mCPT2 in crypts and LPL isolates of *AKR1B8 –/–* colon confirmed the mast cell infiltrations. Mast cell proteases induce tissue injury and permeability, triggering inflammation (Urb and Sheppard, 2012), which supports the fact that *AKR1B8 –/–* mice are susceptible to DSS-induced colitis and colitis associated carcinogenesis (Shen et al., 2015).

Neutrophils and mast cells suppress γδT and CD4T cell function (Kalyan et al., 2014; Sabbione et al., 2014). The γδT cells are critical immune mediators in intestine; mice that lack γδT cells are susceptible to DSS-induced colitis (Kober et al., 2014). Consistently, γδT cells were defective in number and function in the colon of *AKR1B8 –/–* mice that are susceptible to DSS-induced colitis. Through a major histocompatibility complex class II (MHCII)-mediated mechanism, professional antigen presentation cells DCs uptake and present microbial antigens to naïve T lymphocytes, thus initiating adaptive immune response. MHCII deficiency inhibits antigen presentation and leads to immunodeficiency (Chen and Jensen, 2008). To our surprise, total MHCII^+^ DCs decreased in the colon and MLN of *AKR1B8 –/–* mice. Subtype analysis of MHCII^+^ DCs revealed that CD11b^−^ CD103^+^ DCs increased, but CD11b^+^ CD103^−^ DCs decreased in MLN; the subtypes of MHCII^+^ DCs were not altered notably in colon. This is understandable as MHCII^+^ DCs are migratory to flare the adaptive immunity in MLN. Development and maturation of MHCII^+^ DCs and subtypes are multifactorial, including antigen species and concentrations, uptake pathways and cytokine microenvironments (Randolph et al., 2008). Nevertheless, CD11b^−^ CD103^+^ DCs are involved in antigen cross-presentation and priming of CD8T cells (Ruane and Lavelle, 2011); CD11b^−^ CD103^+^ DCs are also a crucial mediator of Treg cells that works in immune tolerance (Coombes et al., 2007). In contrast, CD11b^+^ CD103^−^ DCs are involved in priming of Th1 and Th17 CD4T cells (Liang et al., 2016). Consistently, the IFNγ and IL17-producing CD8T and Treg cells increased, but Th1 and Th17 cells decreased in *AKR1B8 –/–* mice. In summary, in the *AKR1B8 –/–* colon, abnormal innate immune cell infiltration occurred, and γδT cells were dysfunctional and DC development was changed, eventually re-shaping the adaptive immunity.

CD8T cells are cytotoxic T cells (Tc) and function to defense against intracellular pathogens. The increased IFNγ (Tc1) and IL17 (Tc17)-producing CD8T cells may represent a compensatory mechanism to the decreased IFNγ (Th1) and IL17 (Th17)-producing CD4T for pathogen clearance. This makes the colonic immunity at a new balance in *AKR1B8* deficiency, thus being free of spontaneous colitis. However, IL4-producing CD4T and CD8T cells both decreased, which may indicate vulnerability of *AKR1B8 –/–* mice to external stimuli, such as DSS. This is consistent to literature reports that IL-4 knockout mice are susceptible to infection (Noben-Trauth et al., 1996). Treg cells are immune suppressors of effector T cells, i.e., Th1 and Th17 (Paust and Cantor, 2005). In the MLN and colon of *AKR1B8 –/–* mice, Treg cells increased, but functional IL-10 producing Treg cells were not, which may represent a feedback to balance the Treg function in *AKR1B8 –/–* mice.

AKR1B10 is an important regulator of fatty acid/lipid *de novo* synthesis, regulating the PIP_2_ lipid second messenger. Mouse AKR1B8 is the ortholog of human AKR1B10 and mediates fatty acid and lipid synthesis (Joshi et al., 2010). PIP_2_ is a central mediator of AKT and ERK signaling cascades that regulate IECs proliferation, adhesion and integrity (Zhao et al., 2018). NF-κB is a central signaling pathway that regulates cytokine expression. AKT and ERK are involved in the regulation of NF-κB signaling activity via controlling expression and phosphorylation of key effector molecules, such as IKKα and RelA/p65. The decreased p-AKT, p-ERK, p-IKKα/β, p-IKBα and p-p65 (Ser536) in cEC of *AKR1B8 –/–* mice proved the concept that AKR1B8 mediates the PI3K/AKT and PKC/ERK signaling pathways and subsequently the NF-κB activity. AKT and ERK signaling also directly triggers IL-18 expression (Niyonsaba et al., 2005; Venkatesan et al., 2010). Therefore, inhibition of AKT and ERK signaling by AKR1B8 deficiency may induce intestinal immune defects directly or through a NF-κB mediated mechanism. In addition, both AKT (Lee et al., 2010) and ERK (Damiano et al., 2018) signaling regulate Muc2 expression, and accordingly Muc2 was downregulated in the goblet cells of *AKR1B8 –/–* mice and barrier function of colonic epithelium was impaired, which in turn triggered innate immune cell infiltration. It is warranted to dissect in more detail the molecular regulation of cytokine expression and immune response in *AKR1B8 –/–* mice.

**Figure 7:**
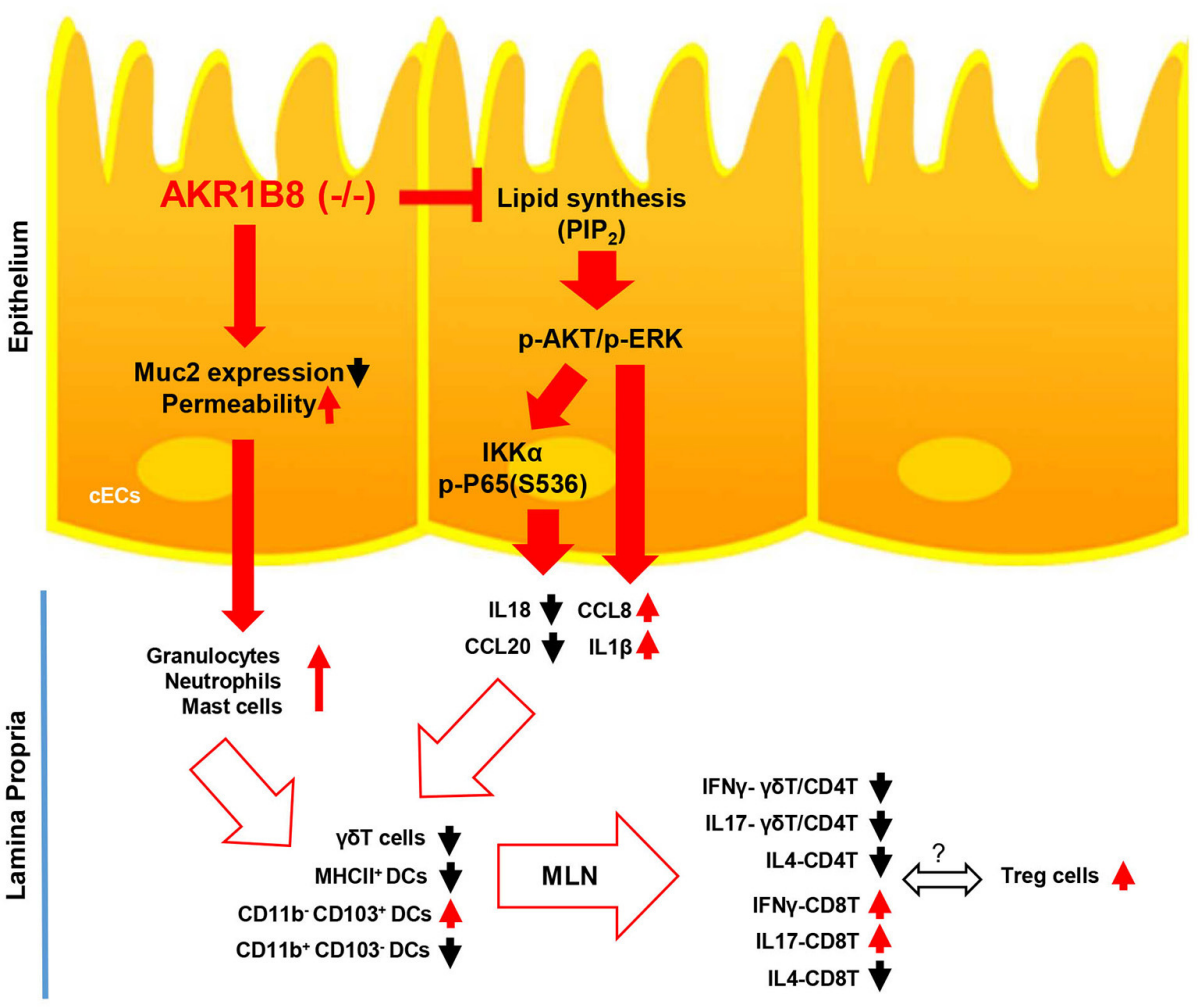
Hypothetic model of AKR1B8 in regulation of intestinal immunity. AKR1B8 deficiency leads to decrease of Muc2 expression and increase of epithelial permeability, triggering inflammatory cell infiltration; AKR1B8 deficiency diminishes synthesis of long chain fatty acid/membrane lipids (e.g., PIP_2_), which attenuates AKT and ERK signaling activity and in turn inhibits NF-κB signaling activity. Diminished AKT, ERK, and NF-κB signaling activity deregulates the expression of immunoregulatory cytokines, which together with infiltrated granulocytes impairs development and function of innate immune γδT cells and antigen presentation dendritic cells, eventually leading to defects of adaptive immune cells.

In conclusion, this study demonstrated the essential role of AKR1B8 in control of intestinal epithelial function and immunity (Figure 7). AKR1B8 deficiency leads to barrier dysfunction and permeability, activating infiltration of granulocytes; AKR1B8 deficiency inhibits AKT and ERK activity and thus disrupts the expression of cytokines, leading to defects of intestinal innate and adaptive immunity. This work unraveled the key role of AKR1B8 in cECs function and intestinal immunity, providing new insights in the regulation of intestinal homeostasis and health.

## Data Availability Statement

The raw data supporting the conclusions of this article will be made available by the authors, without undue reservation.

## Ethics Statement

The animal study was reviewed and approved by Southern Illinois University School of Medicine Laboratory Animal Care and Use Committee.

## Author Contributions

DC and XW designed the research. XW and YS performed animal experiments. XW, YC, and ML performed flow cytometry experiments and analysis. XW, RK, and YZ performed molecular biology experiments. DC and XW wrote the manuscript. D-FL and DC revised the manuscript. All authors contributed to the article and approved the submitted version.

## Conflict of Interest

The authors declare that the research was conducted in the absence of any commercial or financial relationships that could be construed as a potential conflict of interest.
