# Supplementary material for: Impaired Barrier Function and Immunity in the Colon of Aldo-Keto Reductase 1B8 Deficient Mice
Source: Front Cell Dev Biol. 2021 Feb 12;9:632805. doi: 10.3389/fcell.2021.632805 (PMC7907435; doi:10.3389/fcell.2021.632805)
Supplement: Supplementary file 1 [file Data_Sheet_1.PDF]

Fig. S1

a

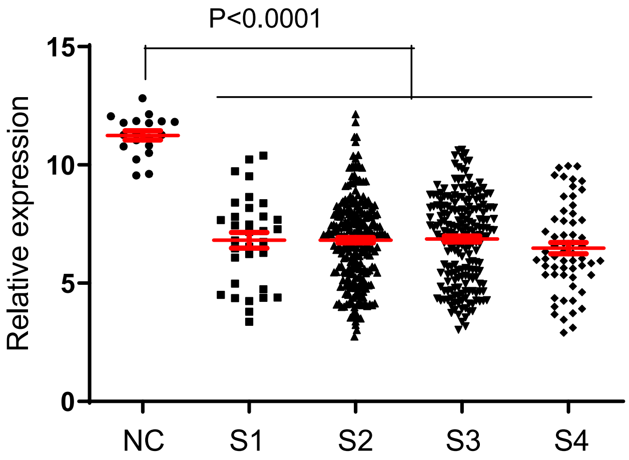

b

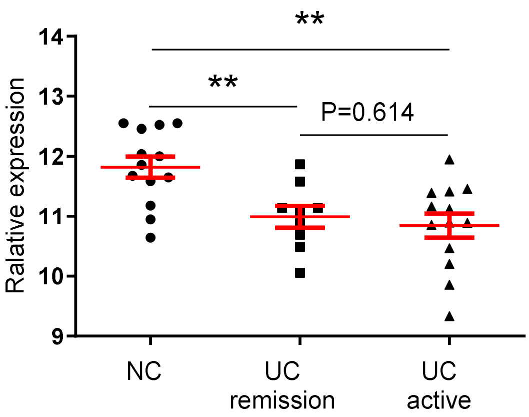

Fig. S1 Expression of AKR1B10 in colorectal cancer, UC and CD tissues. **a** Colorectal cancer. Log2 normalized intensity signal of AKR1B10 expression were plotted. Data were from GSE39582 dataset (n=581). Data show the expression of AKR1B10 in normal controls (NC) and primary colorectal adenocarcinoma at various stages. **b** Ulcerative colitis. Log2 GC-RMA expression value were plotted. Data were from GSE38713 dataset (n=35). Data show the expression of AKR1B10 in normal controls (NC), remitted ulcerative colitis (UC) and active UC are listed. *, $P<0.05$; **, $P<0.01$; ***, $P<0.001$. Statistical significance was tested by unpaired Student's t test.

Fig. S2

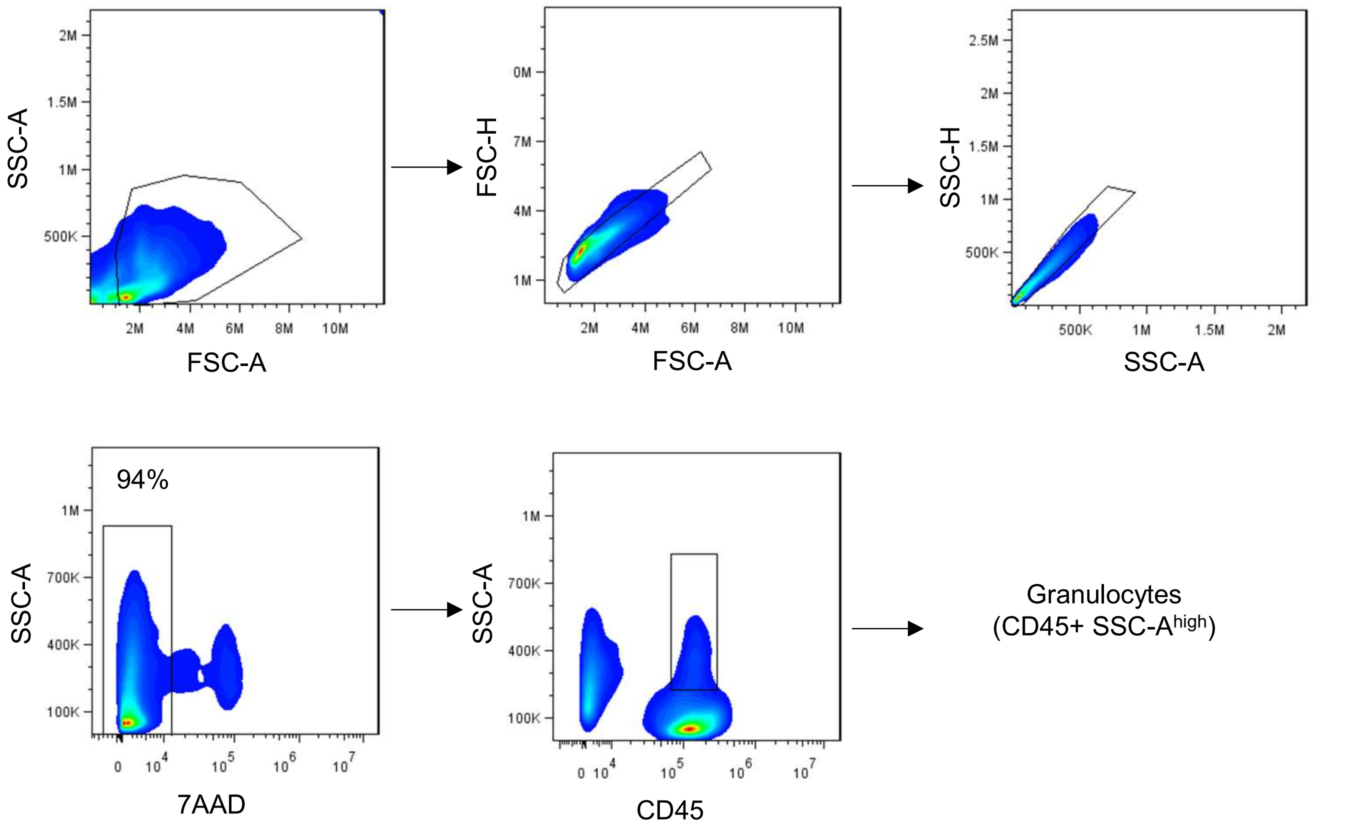

Fig. S2 Gating strategies for lamina propria granulocytes. From left to right: Scatter gate (SSC-A vs FSC-A) was applied to exclude debris; singlets gates (FSC-H vs FSC-A and SSC-H vs SSC-A) were applied to exclude doublets; 7AAD$^-$ gate was applied to gate on live cells (94%); CD45$^+$ gate was applied for leukocytes. Granulocytes were gated as CD45$^+$ SSC-A^{high} leukocytes. FSC, forward light scatter; SSC, side light scatter.

Fig. S3

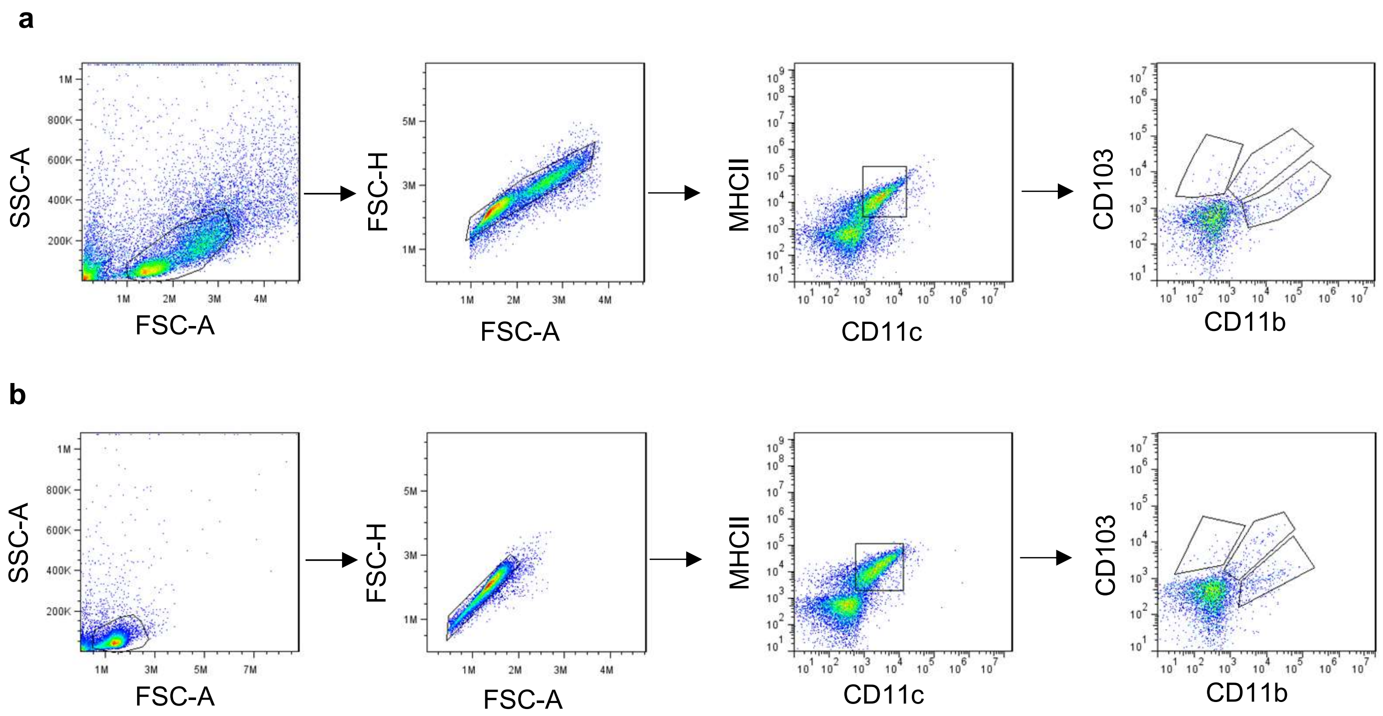

Fig. S3 Gating strategies of MCHII^{+} DCs and DC subsets in colon (**a**) and MLN (**b**). After debris (SSC-A vs FSC-A) and doublets (FSC-H vs FSC-A) were excluded, cells were gated on CD11c^{+} MHCII^{+} DCs and then on CD11b and CD103.

Fig. S4

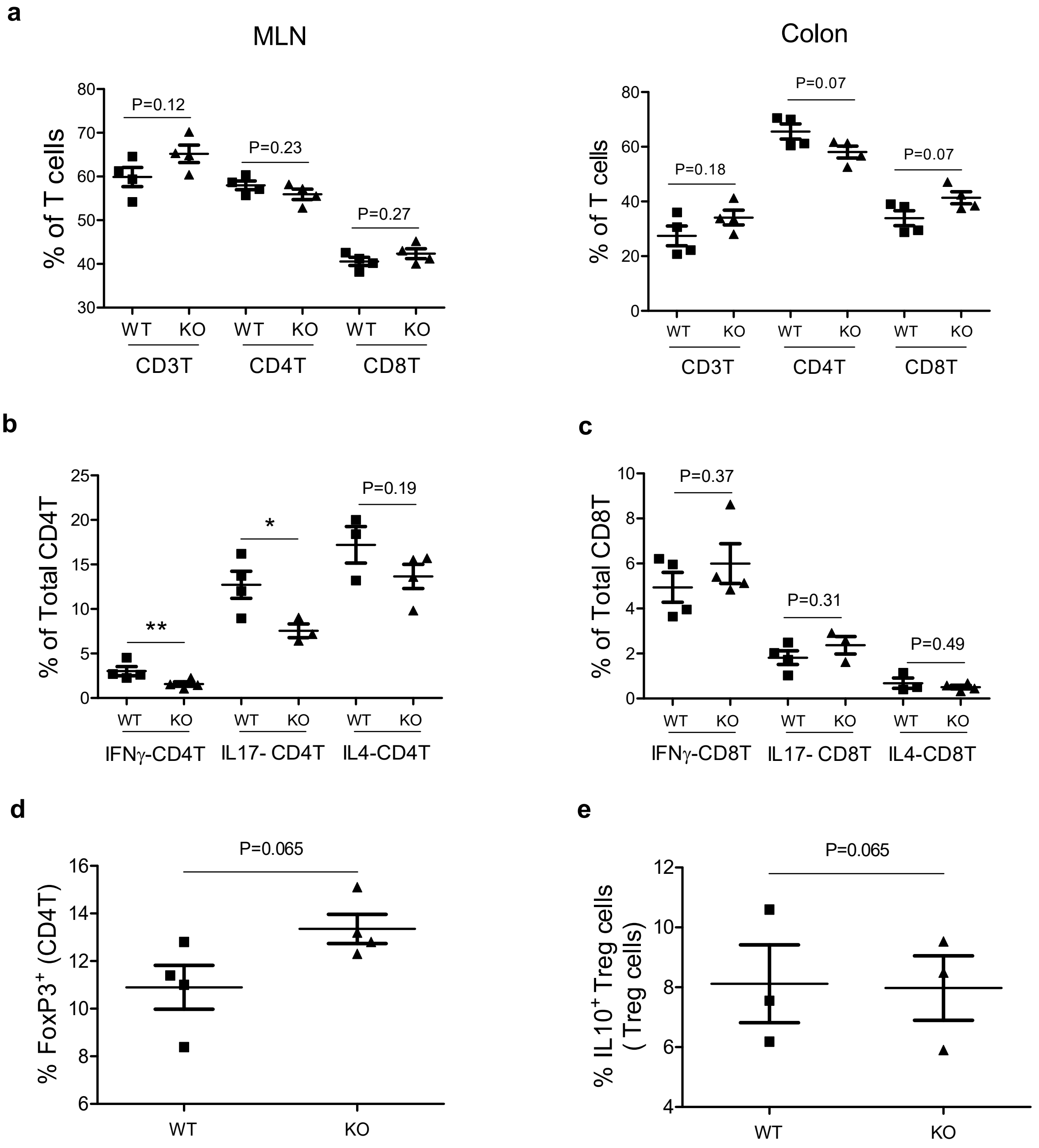

Fig. S4 Adaptive T cells in colon of AKR1B8 -/- mice. **a** CD3T, CD4T and CD8T cell number in MLN and colon of AKR1B8 -/- mice. Data show no notable difference. **b** IFNγ, IL17 and IL4-producing CD4T cells in colon. IFNγ^{+}CD4T and IL-17^{+}CD4T cells decreased in colon of AKR1B8 -/- mice. Data represent mean ± SEM of three to four mice per group. *, $P<0.05$; **, $P<0.01$. **c** IFNγ, IL17 and IL4-producing CD8T cells in colon. Data show no significant difference. **d** CD4^{+} Treg cells in colon. Data show no significant difference. **e** IL10-producing Treg cells. No significant difference.

Supplementary Table 1. Primers used in this study.

Primers	Forward	Reverse
CCL8	TGACTGGGCCAGATAAGGCT	CCATGGGGCACTGGATATTGT
IL22	ATGAGTTTTTCCCTTATGGGGAC	GCTGGAAGTTGGACACCTCAA
IL6	TAGTCCTTCCTACCCCAATTTCC	TTGGTCCTTAGCCACTCCTTC
REG3γ	ATGCTTCCCCGTATAACCATCA	GGCCATATCTGCATCATACCAG
IL1β	GCA ACT GTT CCT GAA CTC AAC	ATC TTT TGG GGT CCG TCA ACT
IL18	TCAGACAACTTTGGCCGACT	CAGTCTGGTCTGGGGTTCAC
S100A8	AAATCACCATGCCCTCTACAAG	CCCACTTTTATCACCATCGCAA
Hamp1	TTGCGATACCAATGCAGAAG	GGATGTGGCTCTAGGCTATGTT
Hamp2	CTGCCTGTCTCCTGCTTCTC	GCAGATGGGGAAGTTGATGT
Pla2g2a	CCACCAAGTTTCCCGGTGAT	GAGTGGTGGGGAAAGGTCAG
Lyz1	GAGACCGAAGCACCGACTATG	CGGTTTTGACATTGTGTTCGC
IL15	ACATCCATCTCGTGCTACTTGT	GCCTCTGTTTTAGGGAGACCT
Mcpt1	TTCACATGGAAAGCCCCCTG	AGAGAGCTCTGGCTTGGAGA
Mcpt2	TTCACCACTAAGAACGGTTCG	CTCCAAGGATGACACTGATTTCA
